# Effects of Konjac Glucomannan on Retrogradation of Amylose

**DOI:** 10.3390/foods11172666

**Published:** 2022-09-01

**Authors:** Shishuai Wang, Lidong Ding, Shuo Chen, Ying Zhang, Jiaxin He, Bin Li

**Affiliations:** 1 College of Food Science and Technology, Wuhan Business University, Wuhan 430056, China; 2 College of Food Science and Technology, Huazhong Agricultural University, Wuhan 430070, China

**Keywords:** konjac glucomannan, amylose, retrogradation, viscoelasticity, texture

## Abstract

The effect of konjac glucomannan (KGM) on the retrogradation of amylose was explored during storage. The color, rheological properties, texture, water-holding capacity (WHC), low-field nuclear magnetic resonance (LF-NMR), and X-ray diffraction (XRD) were investigated. Results of color and rheological measurements showed that with the increasing amount of KGM, the L value of the system decreased, but the elastic modulus, viscous modulus, and tangent value of loss angle increased. The textural result presented that KGM obviously inhibited the growth rate of gel strength of amylose. Results from WHC and XRD suggested after 14 days of storage, when the concentration of KGM increased from zero to 0.3% in the mixture, the WHC grew from 80% to 95% and the crystallinity degree declined from 35.3% to 25.6%. The LF-NMR result revealed that KGM limited the conversion of free water to bound water in the system. In general, a small amount of KGM in a mixed system could inhibit the short-term and long-term retrogradation of amylose. This research could provide a theoretical reference for the influence of hydrophilic colloids on the retrogradation of starch, and it could also provide support for the processing and production of starch-based food.

## 1. Introduction

Starch-based foods are prone to retrogradation during storage, and starch retrogradation is common in food production and processing. Retrogradation can cause the quality of starch-based food to deteriorate, especially in terms of rough taste, hard texture, and loss of food moisture. It finally results in a shortened shelf life of food and a large amount of food waste [1,2].

Amylose, as an important component of starch, is mainly composed of α-1,4 glycosidic bonds. Amylose owns no branch structure, or the distance between branch points is relatively far, exhibiting a linear structure [3]. Due to its linear structure, amylose ages relatively easily. Under the action of hydrogen bonds, amylose aggregates and rearranges to form a double-helix structure, making a cross-networking structure [4,5]. Numerous studies have focused on the regulation of starch retrogradation, and blending with non-starch polysaccharides was found to be one of the effective ways of manipulating starch properties [6].

Konjac glucomannan (KGM) is a neutral heteropolysaccharide derived from the tuber of Amorphophallus konjac. It consists of β-(1,4)-linked D-mannose and D-glucose residues in a molar ratio of 1.6:1 [7]. Because of its large molecular weight and high aqueous solution viscosity, KGM has been approved by the Food Chemicals Codex as a thickener and gelling agent for food production. Moreover, konjac gel as a fat replacer was widely applied to cheese [8,9], yogurt [10], sausages [11], and so on. This kind of food has the advantages of low calories, strong satiety, good taste, and so on, and it is favored by the majority of consumers.

At present, there have been quite a few reports on the influence of KGM on starch retrogradation. Some studies pointed out that KGM could promote the short-term retrogradation of corn starch [12] or the formation of resistant starch [13]. Other studies presented the opposite view that KGM could inhibit starch regeneration to a certain extent, such as in maize starch [14], potato starch [14,15], corn starch [12,16], wheat starch [17], broad bean starch [15], and mung bean resistant starch [18]. In our opinion, whether KGM accelerates or retards starch retrogradation, intermolecular interactions between KGM and starch components, such as amylose, play a crucial role. Many hydroxyl groups exist on the molecular chains of KGM and amylose. The strength of the interaction between the hydroxyl groups is not the same, which might result in different retrogradation behavior of starch [19]. 

Therefore, exploring the effects of KGM on amylose retrogradation is the focus of this research. Different qualities of KGM were added to amylose to prepare a series of blend systems. Then, the color, rheological properties, water-holding capacity, texture properties, moisture distribution, and crystallinity of the system were measured in order to reveal the influence of KGM on the retrogradation properties of amylose. 

## 2. Materials and Methods

### 2.1. Materials

Food-grade konjac glucomannan (KGM) was provided by Hubei Konson Konjac Gum Co., Ltd. (Ezhou, China). Amylose (Amy) derived from corn starch was purchased from Shanghai Yuanye Bio-Technology Co., Ltd. (Shanghai, China). 

### 2.2. Sample Preparation 

Mixtures of amylose (5.0%, *w*/*v*) with KGM (0%, 0.10%, 0.20%, and 0.30%, *w*/*v*) were prepared as follows: KGM powder was primarily dispersed into distilled water and mechanically stirred for 2 h to prepare KGM sol with a constant mass fraction of 1%. Then, amylose (5%, *w*/*v*), KGM sol, and distilled water were blended uniformly to obtain mixtures. After being heated at 100 °C for 30 min, the mixtures were immediately poured into 15 mL breakers with 6 g each. Finally, the mixtures were cooled, sealed, and kept at 4 °C for different storage times.

### 2.3. Color Measurement

Lightness (L), as one of the objective color CIE-LAB tristimulus values, was evaluated on a colorimeter (UltraScan XE HunterLab, Reston, VA, USA). Three determinations were performed for each sample. Before the measurement, the colorimeter was standardized using a white calibration plate.

### 2.4. Dynamic Rheological Measurement

Dynamic rheological experiments were carried out on a controlled-stress Kinexus 2500 rheometer (Malvern Instruments Ltd., Worcestershire, UK) with a cone–plate geometry (CP4/40). After high-temperature gelatinization for 30 min at 100 °C, the above mixtures were immediately tested by time scanning. Silicon oil was applied to the exposed surfaces of samples to prevent evaporation during measurements. Time sweeping was run at shear strain = 1%, frequency = 1 Hz, test time = 5 h, sampling interval = 5 s, temperature = 4 °C.

Dynamic frequency sweeping was conducted from 0.1 and 40 Hz at a constant strain of 1%. The measurements were made at 25 °C. Changes in storage modulus (G′), loss modulus (G″), and loss tangent (Tan δ = G″/G′) were recorded. 

### 2.5. Water-Holding Capacity (WHC)

According to the method of Zhang with some modifications [20], samples were placed in a centrifuge tube (diameter 30 mm) with enough filter paper. Then, the samples were centrifuged for 30 min at 4800 rpm at room temperature. WBC was expressed as the percent of water retained per 100 g of water present in the sample prior to centrifuging. 

### 2.6. Texture Measurement

The gel strength was measured using the compression mode on a TA-XT plus Texture Analyzer (Stable Microsystems Ltd., Surrey, UK). The parameters were as follows: pre-test speed = 1 mm/s, test speed = 1 mm/s, post-test speed = 1 mm/s, compression distance = 4 mm, trigger force = 5 g. After a trigger force was attained, the probe proceeded to penetrate into the gel to a depth of 4 mm. At this depth, the maximum force reading was obtained and translated as gel strength (g). Measurements were performed with a cylindrical probe (P/0.5) at least five times. 

### 2.7. Low-Field Nuclear Magnetic Resonance (LF-NMR)

LF-NMR measurements were performed with an analyzer (MesoQMR23-060H, Niumag Electric Corporation, Shanghai, China). Approximately 2 g of the mixture was placed in a cylindrical glass tube (15 mm diameter) and inserted into the NMR probe. The analyzer operated at 25 °C with a resonance frequency of 21 MHz. The transverse relaxation time (T_2_) was measured using the Carr–Purcell–Meiboom–Gill (CPMG) sequence with the following parameters: SW = 100 kHz, RG = 20 dB, NECH = 18,000, TE = 0.2 ms, NS = 4, TW = 4000 ms. Parameters relating to T_2_ are presented as follows: T_2b_, T_21_, and T_22_ are the relaxation components, while P_2b_, P_21_, and P_22_ are the corresponding area fractions.

In addition, magnetic resonance imaging (MRI) measurements were obtained by MSE sequence with the following parameters: FOV read = 60 mm, FOV phase = 60 mm, averages = 2, TR = 500 ms, TE = 20 ms.

### 2.8. X-ray Diffractometry (XRD)

XRD patterns were obtained for samples with a D/Max-IIIA diffractometer (Rigaku, Tokyo, Japan) using Ni-filtered Cu Kα radiation (λ = 1.54 Å) at 40 kV and 20 mA. The diffraction angle ranged from 5° to 60° with a step-scan of 0.01°. Prior to determination, all samples were lyophilized. Sample crystallinity was determined by plotting the peak baseline on the diffractogram. The areas above and under the curve corresponded to crystalline domains and amorphous regions, respectively. The ratio of upper area to total area was taken as relative crystallinity. 

### 2.9. Statistical Analysis

The data presented are the means and standard deviations determined using the SPSS 19.0 (SPSS Inc., Chicago, IL, USA) for Windows program. Each experiment was measured at least three times unless otherwise stated.

## 3. Results

### 3.1. L Values

After the gelatinization of starch, the rearrangement rate of molecules is a key factor that influences the transparency or the L value of starch paste. If starch molecules do not retrograde easily, they cannot rearrange to form a regular structure. When the light hits the starch paste, no reflection or scattering occurs, and the starch paste has a high transparency or a low value of L [21]. Therefore, there is a close relationship between the L value and retrograding degree of starch. When the retrogradation rate of starch is slow, the transparency of starch paste is high, corresponding to a low value of L. On the contrary, when the retrogradation progress becomes fast, the transparency of starch paste decreases, and the L value rises.

**Figure 1:**
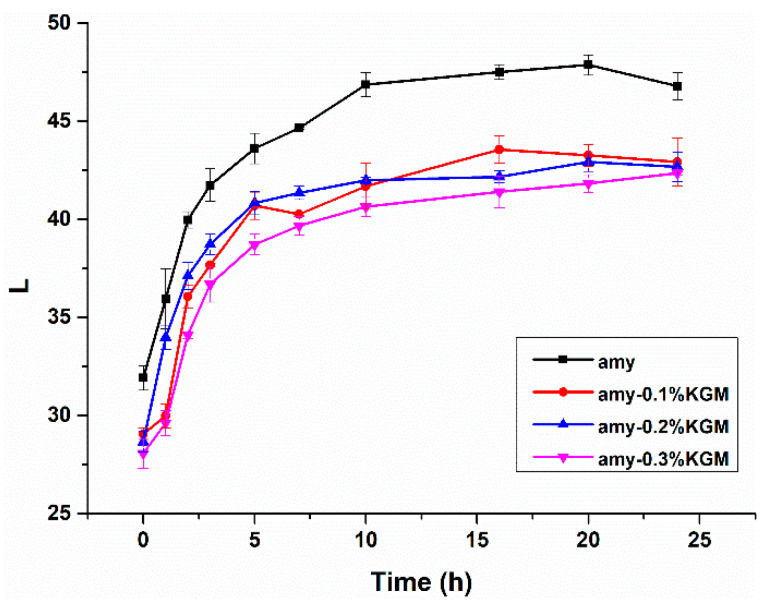
Curves of L values of amylose–KGM mixtures with time.

L values of KGM–amylose mixtures in 24 h after gelatinization can be seen in Figure 1. The L values of all samples gradually increased with time, suggesting that the retrogradation happened. In the first 5 h, the increase in L values was faster. After 5 h, the increase in L values was slower and gradually became stable. This indicated that within 5 h after gelatinization, starch molecules were prone to short-term retrogradation. A higher rearrangement rate was contributed by no branch structure and little steric hindrance of amylose [22]. In addition, at any time the L value of the single amylose was the highest. The addition of KGM led to a decrease in L values. At 0.3% KGM, the L value decreased by approximately 10% compared with that of amylose alone. This phenomenon proved that a small amount of KGM was helpful in delaying the retrogradation or rearrangement of amylose. 

### 3.2. Viscoelasticity

#### 3.2.1. Time Sweeping

**Figure 2:**
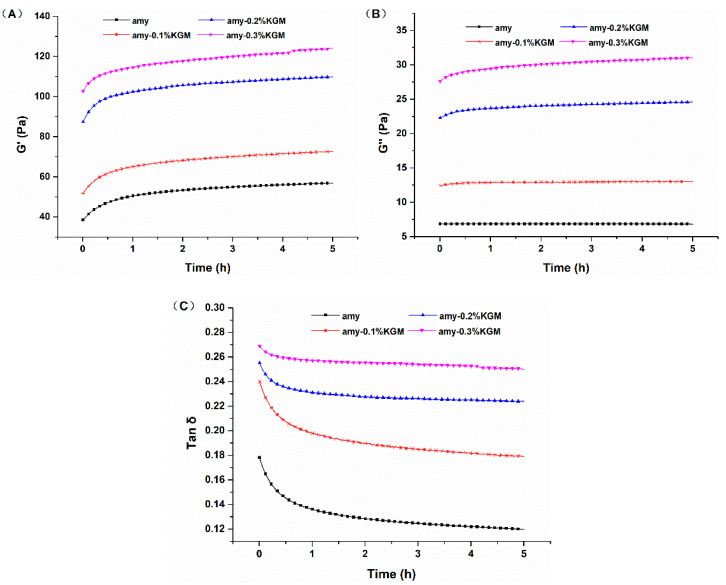
Time sweeping curves of G′ (**A**), G″ (**B**), and Tan δ (**C**) of amylose–KGM mixtures.

To further investigate the influence of KGM on the short-term retrogradation of amylose, after full gelatinization of KGM–amylose mixtures, changes in rheological indexes were monitored immediately at 4 °C within 5 h, including elasticity moduli (G′), viscous moduli (G″), and tangent values of the loss angle (Tan δ). From Figure 2A, it can be seen that the G′ of all samples increased with time. This revealed that amylose molecules were constantly undergoing intermolecular rearrangement and aggregation at low temperature, which resulted in the increasing elasticity of gel structures [23]. In addition, in Figure 2B, the G″ of all samples is rising the whole time, showing that the viscosity of the system continuously strengthened [24,25]. 

Tan δ as the ratio of G″ to G′ was utilized to measure the contribution of viscous properties and elastic properties. The lower the value of Tan δ, the weaker the viscidity and the stronger the elasticity of samples. As Figure 2C shows, Tan δ values of all samples decreased with time. This meant although G′ and G″ from all samples increased, the growth of G′ was greater than that of G″. The retrogradation or rearrangements of all samples were proceeding to different degrees. In addition, Tan δ values of samples blended with KGM were greater than that of the single amylose. As the amount of KGM increased, Tan δ values increased. At 0.3% KGM, G′, G″, and Tan δ were about 1.4, 3.3, and 1.2 times larger than those of single amylose, respectively. The results indicated that KGM in mixtures weakened the relative contribution of elasticity and led to the restriction of the short-term retrogradation of amylose [26]. One reason might be that the interaction between KGM and amylose would promote the formation of a three-dimensional network structure, reflected as the increase in G′. The rising effect of G′ could be enhanced with the increase in KGM concentration. Another reason might be that numerous hydrophilic groups on the molecular chain of KGM could compete to absorb water molecules around the amylose. The extension and entanglement of molecular chains caused the increase in viscosity. On account of the lack of water molecules, the aggregation of amylose was weakened, and the short-term retrogradation of amylose was suppressed.

#### 3.2.2. Frequency Sweeping

**Figure 3:**
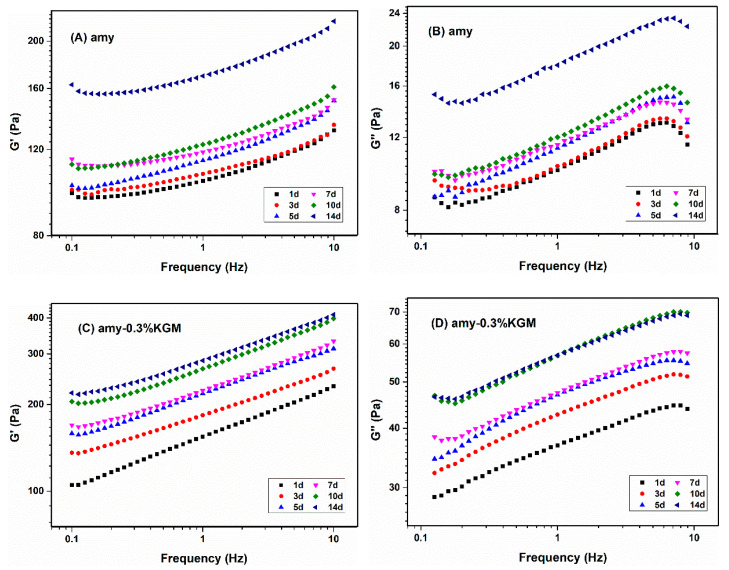
Frequency sweeping curves of amylose and amy-0.3%KGM at different storage times. (**A**) storage modulus of amylose; (**B**) loss modulus of amylose; (**C**) storage modulus of amylose mixed with 0.3% KGM; (**D**) loss modulus of amylose mixed with 0.3% KGM.

In order to explore the influence of KGM on the long-term retrogradation of amylose, the mixtures were stored at 4 °C for a longer time. Figure 3 shows G′ and G″ of single amylose and amylose–0.3% KGM with the change in frequencies at different storage times. It was noted that G′ > G″ for all samples, and G′ and G″ increased with the increase in frequencies, representing a typical weak gel [27]. To further discuss the effects of storage time and KGM amount on the long-term retrogradation of amylose, the power law equation σ = ηω^n^ was fitted, where σ represents shear stress, ω represents the frequency, η represents composite viscosity, and n represents the flow index. The fitting results are shown in Table 1. As the storage time was prolonged from 1 day to 14 days, the composite viscosity of the mixture gradually increased. At the same time, the greater the concentration of KGM in the mixture, the higher the composite viscosity. This suggested that the existence of KGM could change the gel structure of amylose and inhibit the long-term retrogradation of amylose.

### 3.3. Gel Strength

**Figure 4:**
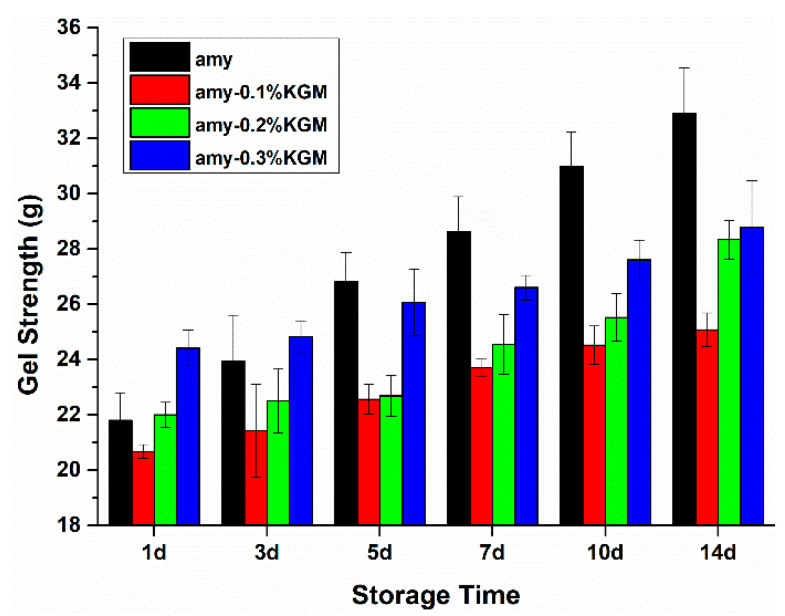
Gel strength of amylose–KGM mixtures at different storage times.

After being gelatinized and refrigerated, the starch molecules would rearrange to form a gel. Gel strength could indirectly reflect the aging degree of starch. In general, the greater the gel strength, the higher the retrograding degree of starch. Changes in gel strength of KGM–amylose mixtures with time are presented in Figure 4. With the storage time extended to 14 days, the gel strength of all samples showed an increasing trend, indicating that the amylose molecules in each sample were constantly rearranging, and the retrogradation of different samples was proceeding to different degrees. At the same time, when the mass fraction of KGM in the mixture increased from 0.1% to 0.3%, the gel strength of the sample gradually increased, indicating that the addition of KGM could increase the structural tightness of the amylose system, leading to the increase in gel strength.

At a shorter storage time, such as one day, when the mass fraction of KGM reached 0.2–0.3%, the gel strength of the samples was higher than that of the pure amylose. On the third day, when the mass fraction of KGM was 0.3%, a similar phenomenon appeared. However, after five days, the gel strength of samples with KGM began to be lower than that of amylose alone. It could be supposed that the single amylose had the largest increase rate of gel strength or the highest degree of retrogradation after the entire storage period. The reason might be the case that on the one hand, KGM could interact with amylose, resulting in the increase in structural tightness of gel. On the other hand, the existence of KGM could also interfere with the ordered rearrangement of amylose to some extent, leading to a lower increase rate of gel strength or a smaller degree of retrogradation [28].

### 3.4. WHC

**Figure 5:**
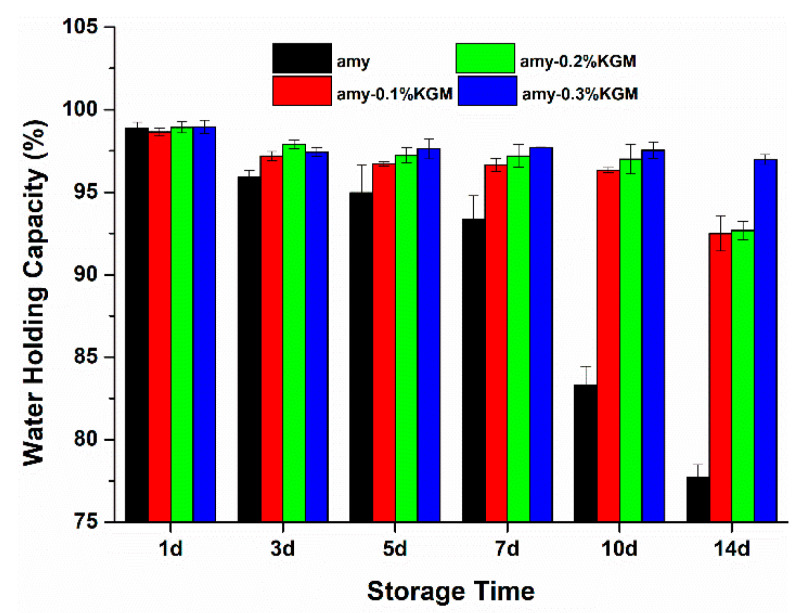
Water-holding capacity of amylose–KGM mixtures at different storage times.

The WHC changes of KGM–amylose samples during storage are shown in Figure 5. With the extension of time, the WHC of all samples showed a downward trend, especially in the single amylose, indicating that starch retrogradation occurred in different degrees. At the storage of 14 days, the WHC of single amylose fell sharply to less than 80%, but the WHC of samples blended with KGM remained above 95%. In addition, during the entire storage, the WHC of all samples increased with the increase in the amount of KGM. This showed that with the help of KGM, starch paste could form a network structure and retain water molecules well, allowing the degree of starch retrogradation to be inhibited. Similar results have been reported in a study showing that the addition of 0.3–0.5% pullulan contributed to maintaining the integrity of rice starch gel, preventing water precipitation from the gel, and inhibiting the retrogradation and structural deterioration of rice starch during cold storage [29]. The Mesona chinensis polysaccharide could decrease the syneresis rate of starch, and the phenomenon became more obvious as the polysaccharide concentration increased. This indicated that the MCPS could strengthen the gel network of starch [30,31].

### 3.5. Water Distribution

**Figure 6:**
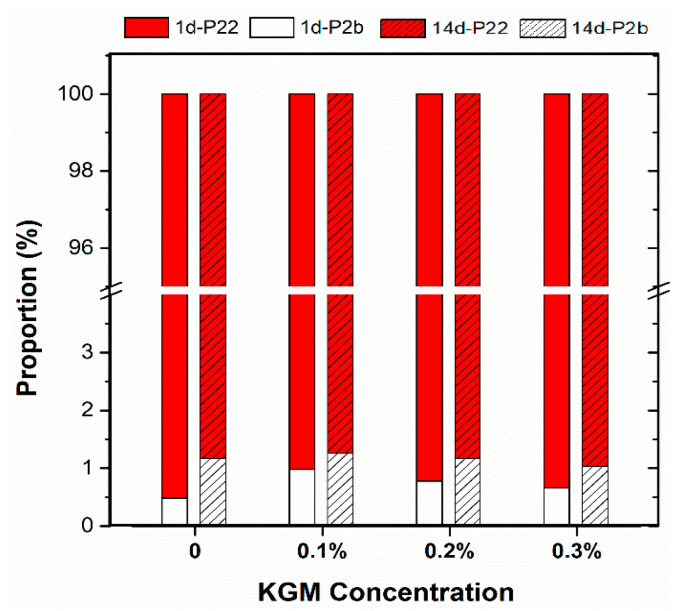
The proportion of water distribution of amylose–KGM mixtures at different storage times. (1d-P22 and 1d-P2b represent the proportions of free water and bound water in the mixtures after 1 day of storage, respectively; 14d-P22 and 14d-P2b represent the proportions of free water and bound water after 14 days of storage, respectively).

LF-NMR was adopted to measure the water statuses of the gel. The water in the system could be commonly divided into three statuses: bound water, not easily flowing water, and free water. Their transverse relaxation time was distributed at 0.1–10 ms, 10–100 ms, and 100–1000 ms in turn [32]. In the KGM–amylose mixtures, the peak positions of all samples appeared around 1 ms and 1000 ms, indicating that there were only two statuses of water in the mixed system, which were bound water and free water. The proportion of water in different statuses was calculated by the peak area, and the result is revealed in Figure 6. After the storage of 14 days, the tightly bound water in each sample increased, while the free water decreased. Starch retrogradation required the participation of water, and the free water in the system was converted into bound water. Along with the amylose molecular rearrangement and the reformation of crystal structure, water was tightly forced into amylose chain segments [33,34]. 

In addition, it was found that the larger the concentration of KGM, the higher the freedom of most water in the system, and the less conversion of free water to bound water. Schwartz pointed out that 1% KGM could slightly decrease the amount of available water in a starch suspension, preventing the gelatinization and retrogradation of starch [15].

**Figure 7:**
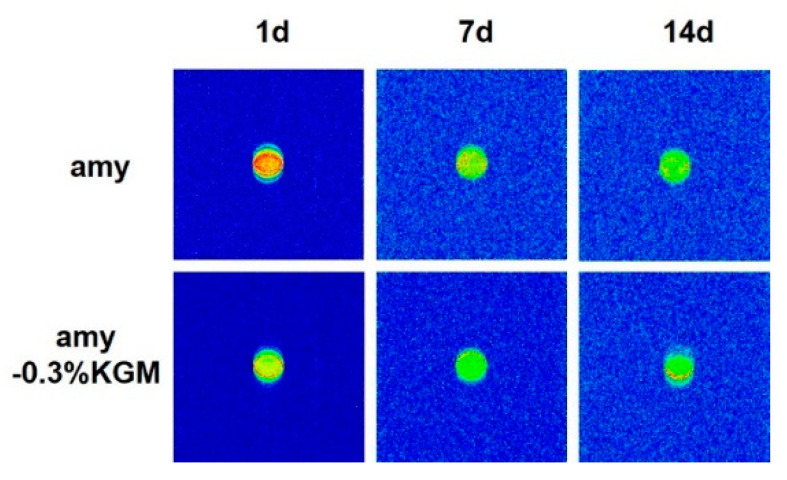
Water distribution of amylose–KGM mixtures at different storage times.

Moreover, water distribution in the mixed system is shown in Figure 7. The density of hydrogen protons in the sample represents the number of hydrogen protons. The higher the hydrogen proton density, the higher the water content, and the deeper the red color in the pseudo-color image [35]. As shown in Figure 7, water distribution in pure amylose was relatively equal. When KGM was added to amylose, water distribution became unequal, and water molecules focused on the outside of the starch paste. This might be explained by the strong hydrophilicity of KGM molecules, which could inhibit water from entering the starch molecules and hinder the retrogradation process of starch [36].

### 3.6. Crystallinity

**Figure 8:**
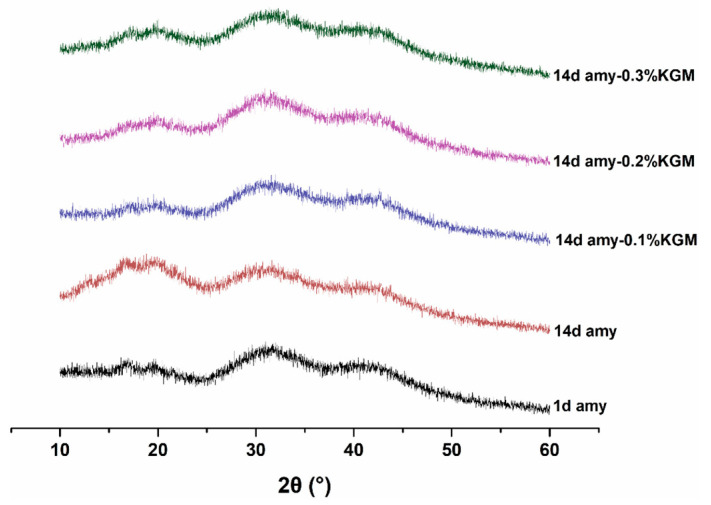
XRD curves of amylose–KGM mixtures at different storage times.

XRD is an efficacious means of measuring crystal structure, and it can reflect the retrogradation degree of starch by calculating the relative crystallinity. As shown in Figure 8, the diffraction peaks of the samples appeared around 2θ angles of 17°, 20°, 31°, and 41°, displaying a B+V-type crystal structure. It had been reported that 17° and 20° were the typical diffraction peaks of type B and type V starch, respectively [18].

For a single component of amylose, when the storage was extended from 1 day to 14 days, the intensity of each diffraction peak increased; meanwhile, the relative crystallinity improved from 31.0% to 35.3%. This indicated that the retrogradation of amylose was happening. For the mixture of KGM–amylose, the shape and position of each peak almost did not change. When the mass fraction of KGM was 0.1%, 0.2%, and 0.3%, the relative crystallinity of the mixed system was 25.6%, 25.0%, and 24.1%, respectively. This suggested that with the increase in the amount of KGM, the relative crystallinity of the mixtures decreased slightly. However, compared to that of pure amylose, the crystallinity of the mixtures was reduced obviously. This proved again that the existence of a small amount of KGM could effectively inhibit the retrogradation of amylose [37].

## 4. Conclusions

Different amounts of KGM were added to amylose paste in order to investigate the effect of KGM on the retrogradation behavior of amylose in the short and long term. Compared with pure amylose, the mixture of KGM–amylose exhibited a decreased L value of the system and an increased elastic modulus, viscous modulus, and tangent value of loss angle. At 0.3% KGM, the L value of the system was approximately 10% lower and the elastic modulus, viscous modulus, and tangent value of loss angle were about 1.4, 3.3, and 1.2 times larger than those of single amylose, respectively. These results indicated that KGM could obviously delay the short-term retrogradation process of amylose. In addition, the existence of KGM also inhibited the growth rate of gel strength, improved the water-holding capacity, and reduced the crystallinity of mixtures. When KGM concentration grew from zero to 0.3% in the mixture, the WHC grew from 80% to 95% and the crystallinity degree was reduced from 35.3% to 25.6% after 14 days of storage. It was speculated that on the one hand, the interaction between KGM and amylose prevented molecules near each other from forming an orderly double-helix structure. On the other hand, due to the strong hydrophilicity of KGM, the transformation of free water to bound water in the mixture was limited. The participation of water molecules involved in the rearrangement of amylose was prevented, thus delaying the long-term retrogradation behavior of amylose. This study could provide a reference for the application of hydrophilic colloids such as KGM in the processing of starch-based foods, such as noodles, bread, steamed bread, and biscuits. 

## Figures and Tables

**Table 1:** Composite viscosity amylose–KGM mixtures fitted by the power law equation.

Time (days)	Amy (Pa·s)	Amy-0.1%KGM (Pa·s)	Amy-0.2%KGM (Pa·s)	Amy-0.3%KGM (Pa·s)
1	0.962	1.104	1.167	1.157
3	0.994	1.107	1.175	1.441
5	1.031	1.118	1.281	1.743
7	1.088	1.295	1.319	2.057
14	1.521	1.596	1.805	2.233

## Data Availability

Data is contained within the article.
